# Leptin and Leptin Signaling in Multiple Sclerosis: A Narrative Review

**DOI:** 10.1007/s12017-025-08842-4

**Published:** 2025-02-28

**Authors:** Juan Antonio Flores-Cordero, Amalia Aranaz-Murillo, Teresa Vilariño-García, Antonio Pérez-Pérez, Guillermo Izquierdo, Rocío Flores-Campos, Lourdes Hontecillas-Prieto, Daniel J. García-Domínguez, Víctor Sánchez-Margalet

**Affiliations:** 1 Department of Medical Biochemistry and Molecular Biology, and Immunology, Medical School, University of Seville, Seville, Spain; 2 Department of Medical Biochemistry and Molecular Biology, and Immunology, Medical School, Virgen del Rocio University Hospital, Seville, Spain; 3 Neurology Service, Virgen Macarena University Hospital, University of Seville, Seville, Spain; 4 Department of Clinical Oncology, Hospital Universitario Virgen Macarena, University of Seville, Seville, Spain; 5 Clinical Biochemistry Service, Hospital Universitario Virgen Macarena, University of Seville, Seville, Spain; 6 Institute of Biomedicine of Seville, IBiS/Virgen del Rocío-Virgen Macarena University Hospital/CSIC/University of Seville, Seville, Spain; 7 Department of Medical Biochemistry and Molecular Biology, and Immunology, Medical School, Virgen Macarena University Hospital, University of Seville, Av. Sánchez Pizjuan 4, 41009 Seville, Spain

**Keywords:** Leptin, Obesity, Multiple Sclerosis, Inflammation

## Abstract

Obesity, a pandemic health problem, is now considered as a chronic inflammatory state, related to many autoimmune diseases, such as multiple sclerosis. Thus, adipokines, inflammatory mediators secreted by adipose tissue, play an important role modulating the immune response. In this context, obesity, especially during adolescent age, seems to be a key factor for the development of multiple sclerosis. Leptin, the main pro-inflammatory adipokine secreted by the adipose tissue, has been found increased in patients with multiple sclerosis and is able to regulate the immune system promoting a pro-inflammatory response. Leptin signaling in both innate and adaptative immune cells might have immunomodulatory effects in the context of multiple sclerosis. In this way, leptin has been found to produce a Th1 and Th17 response, increasing M1 macrophages and decreasing regulatory T cells and Th2 response. Moreover, circulating inflammatory adipokines, such as leptin, have been found in people with multiple sclerosis. In the present work, we are reviewing literature to update the body of knowledge regarding the role of obesity and leptin in multiple sclerosis.

## Introduction

Multiple Sclerosis (MS) stands as the most prevalent autoimmune demyelinating disease affecting the central nervous system (CNS), marked by inflammation, selective myelin destruction, and gliosis, ultimately leading to neuronal loss. Initially published in 2008 through a collaborative effort between the MS International Federation (MSIF) and the World Health Organization (WHO), the first edition aimed to determine the global prevalence of MS. Subsequently, in a revised second edition, the MS International Federation reported a rise in diagnosed people with MS from 2.3 million in 2013 to 2.8 million in 2020 and 2.9 million in 2023. This increase can be attributed to enhanced MS diagnosis, improved treatment and support, and enhanced capabilities in tallying individuals with MS in Northern Hemisphere countries, including the USA, Canada, and Europe (The Multiple Sclerosis International Federation, Atlas of MS, 3rd Edition, September 2020; www.atlasofms.org). Moreover, shifts in lifestyle habits, such as western diets and sedentarism, may contribute to the observed increase (Dargahi et al., 2017). MS exhibits a higher incidence in women than men, ranging from 2:1 to 3:1 depending on the source. More concretely, for RRMS it is 3:1, whereas for PPMS it is 1:1 (Jakimovski et al., 2024). While MS can manifest at any age, it is more frequently observed between 20 and 40 years old, making adolescence a critical period for potential disease development (Dargahi et al., 2017), Consequently, attention is warranted toward potential risk factors, such as obesity, that may increase disease incidence during adolescence, even though only 3–5% of all individuals diagnosed with MS experience disease onset before 16 years of age.

The presentation of MS varies widely among patients, ranging from sudden onset to insidious or slow progression with diverse symptoms. Four main variants have been delineated based on disease evolution: relapsing/remitting (most of the cases), secondary progressive, primary progressive, and progressive relapsing (Dargahi et al., 2017). The relapsing/remitting variant appears to have a more pronounced immune component contributing to MS pathology (Baecher-Allan et al., 2018). The advent of magnetic resonance imaging (MRI) has revolutionized both the diagnosis and treatment of MS. The use of gadolinium as intravenous contrast facilitates the early detection of inflammatory lesions, particularly in the relapsing variant, which often occurs in the initial phase of MS development. However, the diagnostic efficacy of gadolinium is less robust in progressive forms of the disease (Baecher-Allan et al., 2018). Additional diagnostic tools such as evoked potentials and cerebrospinal fluid (CSF) examination can be valuable. Mononuclear pleocytosis, elevated IgG concentration, and the presence of oligoclonal bands support the diagnosis of MS.

Despite ongoing research leading to advancements in MS management, there is currently no effective treatment promoting remyelination or neural repair. Contemporary treatments aim to suppress the immune system (e.g., glatiramer acetate, IFN-beta, natalizumab, fingolimod, and alemtuzumab, among others). While the prognosis of the disease has improved, a significant proportion of people with MS ultimately experience progressive neurological disability.

**Fig. 1:**
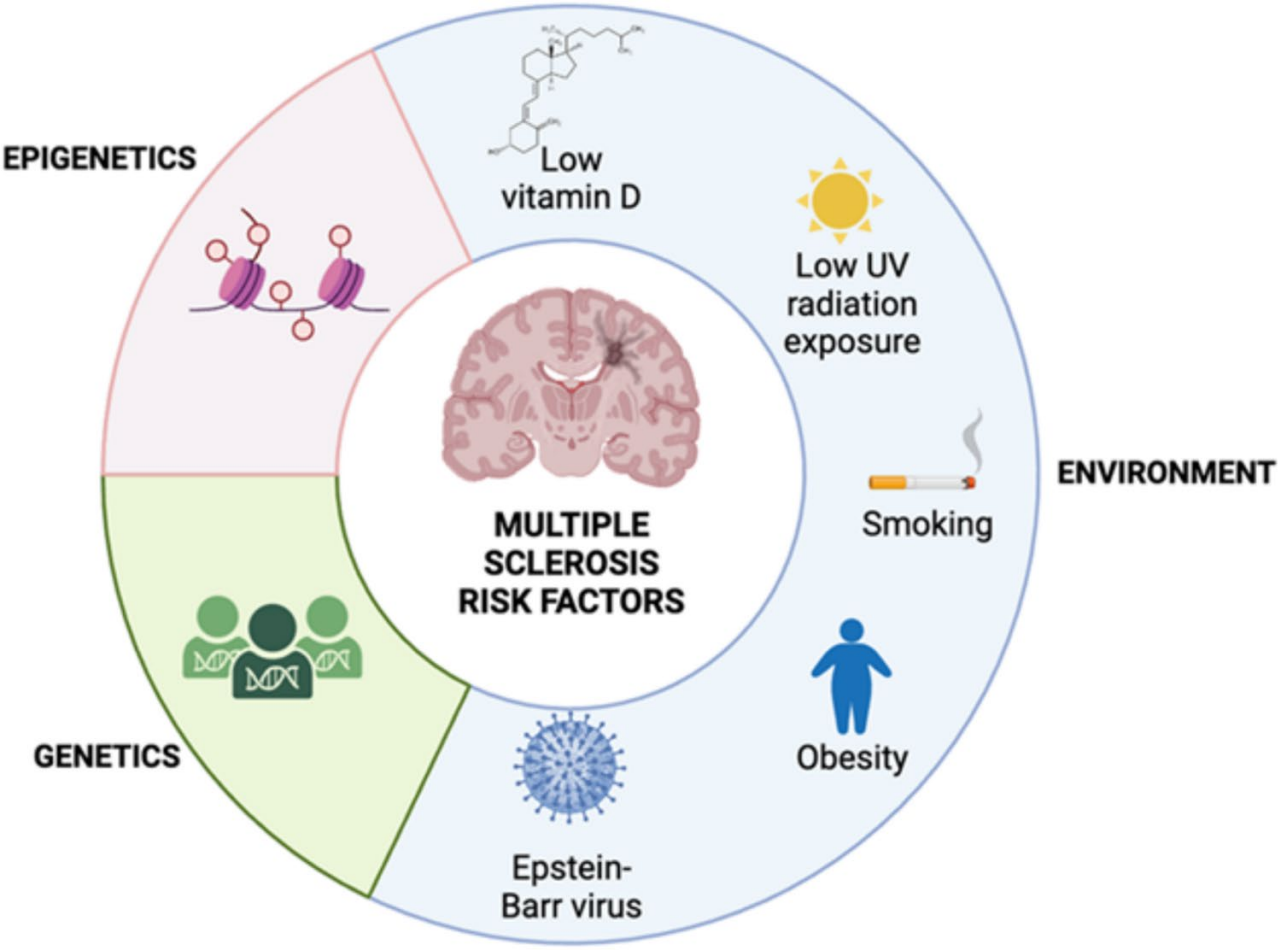
Risk factors of multiple sclerosis

Like many other autoimmune diseases, multiple sclerosis (MS) is a multifactorial condition influenced by various risk factors, encompassing environmental, genetic, and epigenetic elements (see Fig. 1). Despite numerous proposed antigens, none have been conclusively confirmed as the definitive cause of the disease. Presently, obesity is recognized as a pro-inflammatory state and is considered a potential risk factor for various inflammation-related conditions, including diabetes, cardiovascular disease, cancer, and autoimmune diseases, such as multiple sclerosis (Kinlen et al., 2018). Adipokines, including leptin—a major adipokine produced by adipose tissue—may contribute to the complications of obesity due to their pro-inflammatory actions (Pérez-Pérez et al., 2017, 2020a; Sánchez-Margalet et al., 2010). However, it is hypothesized that a dysregulation in adaptive immune response, coupled with a pro-inflammatory environment, plays a pivotal role in mediating the disease process.

In this context, with a focus on obesity as a potential risk factor for multiple sclerosis (MS), the objective of this article is to comprehensively review the literature pertaining to the roles of obesity and leptin in the development of MS.

### Pathophysiology

To direct this review, we will focus on MS as an autoimmune disease influenced by environmental factors acting upon a genetically susceptible host (Baecher-Allan et al., 2018). The disease is characterized by inflammation, selective myelin destruction, and gliosis, eventually leading to neuronal death. In the contemporary understanding of MS pathophysiology, the adaptive immune system is recognized to play a significant role.

A distinctive pathological feature of MS is the presence of perivenular inflammatory lesions, giving rise to demyelinating plaques that precede axon degeneration (Kornek & Lassmann, 1999; Kutzelnigg et al., 2005; Lucchinetti et al., 2011; Fisniku et al., 2008; Lemus et al., 2018; Trapp et al. 1999).

This response triggers reactions against myelination, involving various immune cells, such as CD4 + and CD8 + T cells, B cells, or natural killer (NK) cells. Additionally, inflammatory lesions reveal the presence of other cell types, including microglial cells and infiltrated macrophages, forming a narrow rim around the inflammatory site (Prineas et al., 2001). Thus, both adaptive and innate immune systems are implicated in the complex pathophysiology of MS.

The subsequent sections will delve into the various events occurring in MS, encompassing immunity and inflammatory responses, demyelination, and axonal degeneration.

Reduced vitamin D levels, diminished sun exposure, cigarette smoking, obesity, and exposure to the Epstein–Barr virus (EBV) appear to be environmental risk factors for MS (Baecher-Allan et al., 2018) (Fig. 1). Consequently, our focus will be on obesity as a potential risk factor for MS, as discussed in the following section and below.

## Obesity and Multiple Sclerosis

Obesity can be considered as a state where an excessive body mass index exists. An alteration in energy balance between intake and expenditure is produced during an obesity state. The major factor that causes energy expenditure is exercise. Nevertheless, exercise accounts for only 20–30% of energy expenditure in the sedentary lifestyle of Western societies, whereas 70–80% corresponds to basal metabolism. According to the WHO, 35% of the global population exhibits an increased body mass index (BMI), falling into the categories of overweight (> 25 kg/m^2^) or obesity (> 30 kg/m^2^) (Versini et al., 2014). Moreover, a rise in adipose tissue has been correlated with cognitive dysfunction (de Candia & Matarese, 2018; Flores-Cordero et al., 2022). A substantial body of compiled evidence supports the association between metabolic changes, such as obesity, and neurodegeneration in various neurological disorders, including multiple sclerosis (MS), owing to chronic neuroinflammation. Indeed, the epidemiological link between obesity and neurodegeneration has been validated in diverse animal models (de Candia & Matarese, 2018; Procaccini et al., 2016).

Several immune system-related diseases have been connected to obesity. Aggravated forms of autoimmune diseases have been identified in obese individuals, exhibiting a weaker therapeutic response (Bapat et al., 2022; Kvistad et al., 2015). Elevated BMI has been implicated in an increased risk of developing MS (Gianfrancesco et al., 2014; Hedström et al., 2012; Høglund et al., 2021; Marrodan et al., 2021), particularly when this elevation occurs during adolescence (Chitnis et al., 2016; Hedström et al., 2015; Høglund et al., 2021; Huppke et al., 2019; Munger et al., 2009). This increased risk of MS suffering has been linked to obesity in the adolescence; moreover, obesity worsen the first-line treatment efficacy, since a higher percentage of adolescent obese people with MS must follow to a second-line treatment because the disease-modifying drugs (interferon beta-1a or 1b and glatiramer acetate) were less effective in obesity (Huppke et al., 2019). Owing to that reason factors such as obesity must be taken in account when first-line treatment is administered, because obesity can condition the therapy outcomes. Additionally, the heightened risk for MS development appears to be more pronounced in females than in males (Gianfrancesco et al., 2014; Langer-Gould et al., 2013; Munger et al., 2013) suggesting a gender-specific effect.

**Fig. 2:**
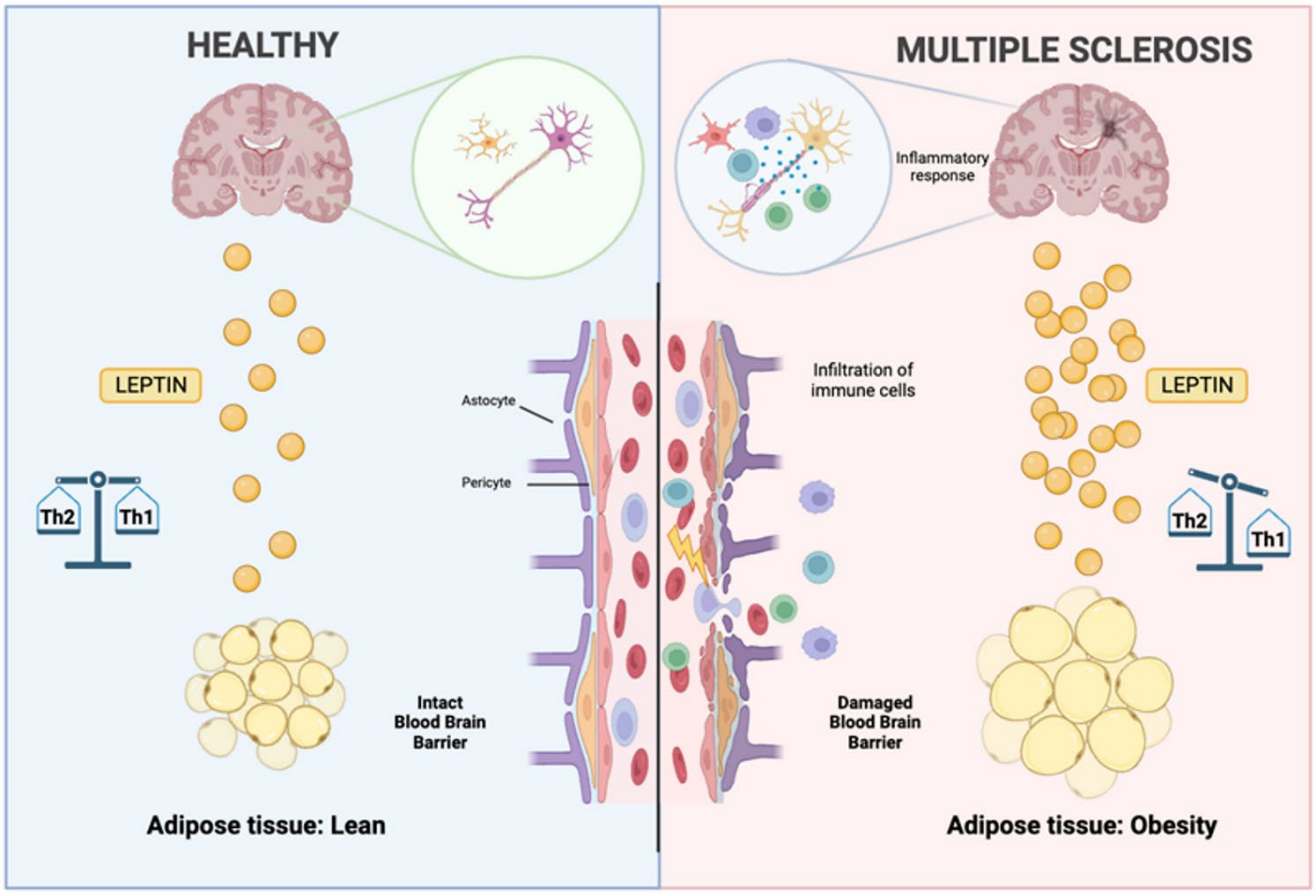
There seems to be a balanced immune state in lean subject with an intact blood–brain barrier (BBB) and a healthy central nervous system, whereas there seems to be a chronic low-grade inflammation state in obese individuals with a compromised BBB because of the disbalanced inflammatory state present in these subjects. This situation may facilitate the infiltration of immune cells into the brain parenchyma of obese individuals. Obesity-induced inflammation may be a contributing factor for MS, in which glial cells are attacked, leading to myelin loss and neurodegeneration. Leptin is a pro-inflammatory adipokine secreted in higher quantities by obese people’s adipose tissue

A proposed initializing mechanism for various neurodegenerative diseases, such as Alzheimer’s Disease or MS, is a low-grade inflammatory state associated with obesity (Correale & Marrodan, 2022; Flores-Cordero et al., 2022; Marrodan et al., 2021; Samara et al., 2023) (Fig. 2). Notably, an anti-inflammatory environment appears to prevail in the adipose tissue of lean individuals, whereas a pro-inflammatory environment characterizes the adipose tissue of obese individuals. In lean adipose tissue, there is an anti-inflammatory profile marked by the presence of regulatory T cells (Treg cells), natural killer (NK) cells, invariant NK (iNKT) cells, M2 macrophages, innate lymphoid cells type 2 (ILC2), and eosinophils. Conversely, obese adipose tissue exhibits a pro-inflammatory profile with an increased presence of M1 macrophages, neutrophils, CD8 + T cells, T helper 1 (Th1) cells, and a decreased presence of iNKT cells, ILC2 cells, and Treg cells, as well as Th2 immunosuppressive mediators (e.g., Interleukin-4 (IL-4), IL-10, Transforming Growth Factor (TGF-β)). This imbalance results in a local and systemic dysregulation of the immune system, leading to an inflammatory chronic low-grade state that is believed to be transferred to the central nervous system (CNS), potentially exacerbating MS (Davanzo et al., 2023). Accordingly, studies realized in animal models show that a systemic chronic low-grade inflammation originating in adipose tissue may impact the blood–brain barrier (BBB), causing a reduction in transcytosis and tight junction proteins in animal models (Pfeiffer et al., 2011; Ransohoff et al., 2015; Varatharaj & Galea, 2017). This, coupled with inflammation-induced upregulation of vascular cell adhesion molecule 1 (VCAM-1), intercellular adhesion molecule 1 (ICAM-1), P-selectin, and E-selectin, could induce leukocyte extravasation (Carvalho-Tavares et al., 2000; Chai & Song, 2014). Additionally, both Tumor Necrosis Factor (TNF)-α and IL-1β induce the expression of the chemokines chemokine (C-X-C motif) ligand (CXCL)1 and CCL2, further enhancing leukocyte recruitment (Skelly et al., 2013). These processes indicate possible translation of immune phenomena from adipose tissue to the brain, which might induce pathological events, such as MS.

The onset of MS during pediatric ages is associated with greater disability and disease severity compared to adult-onset MS in epidemiological studies (Pétrin et al., 2018). While the early occurrence of MS or its precursor, clinically isolated syndrome, was thought to be rare, its incidence is increasing (Langer-Gould et al., 2013). In fact, 3–5% of adults with MS have been found to exhibit clinical symptoms before the age of 18 (Gianfrancesco et al., 2014). As highlighted earlier, MS is a multifactorial disease involving both genetic and environmental factors (Pétrin et al., 2018). Consequently, the question arises: could obesity during pediatric age be a risk factor for MS? Several studies suggest a positive response to this question (Pétrin et al., 2018; Sikes et al., 2018). However, moderate obesity in preadolescent age (5–10 years) does not appear to be associated with the risk of developing MS. On the other hand, a population based studied has concluded that obesity in adolescence and young adulthood increases 50% the risk of MS (Høglund et al., 2021). Regarding the association between body mass index (BMI) and MS progression, published studies present inconclusive and contradictory findings. No statistically significant association seems to exist between BMI and the presence of oligoclonal bands in cerebrospinal fluid, disease duration, and expanded disability status score (EDSS) (Çoban et al., 2015). However, it has been observed that overweight or obese individuals respond less favorably to interferon-beta treatment. Additionally, some studies suggest that obese and smoking patients face a higher risk of disease progression compared to obese non-smokers, with smoking acting as a potential confounding factor (Bove et al., 2016).]. Recently, epidemiological data seem to associate obesity with higher disease severity and poorer outcome (Lutfullin et al., 2023) and together with other risk factors such as smoking may contribute to early disease activity (Briggs et al., 2018).

Gender differences have been noted in the association between high BMI and increased disability over the years. In obese women, a higher BMI is associated with a worse EDSS, while in men, contrary to expectations, overweight or obesity is linked to a lower EDSS (Bove et al., 2016). However, some studies have only found a statistically positive association between high BMI and disease progression in men (Paz-Ballesteros et al., 2017). A potential explanation for these results is the differing body composition, with variations in adipose tissue and musculoskeletal tissue depending on gender (Bove et al., 2016).

Therefore, adopting healthy lifestyle habits such as not smoking and avoiding alcohol could potentially improve the prognosis of patients with MS (Paz-Ballesteros et al., 2017). An interesting study suggests that people with MS do not tend to gain weight as the years pass, unlike the healthy population. This phenomenon can be explained by two theories. Firstly, after years following the onset of the first symptoms, most patients are already overweight or obese. The second explanation stems from the progressive sarcopenia occurring in these individuals due to inflammation and degenerative processes in the disease (Bove et al., 2016).

## Pro- and Anti-Inflammatory Effects of Adipokines

Both food intake and energy expenditure are regulated by the CNS, which integrates peripheral signals such as hormones, metabolic mediators, and signals from the peripheral nervous system. These signals act through the sympathetic and the parasympathetic nervous systems to control basal metabolism.

The first identified cause of monogenic obesity was a deficiency in leptin, a hormone that regulates food intake and basal metabolism at the central level (Zhang et al., 1994). Administration of leptin has been shown to prevent obesity in individuals with this deficiency (Halaas et al., 1995; Pelleymounter et al., 1995).

Nowadays, adipose tissue is considered not only a “lipid store,” but also an endocrine organ (Kershaw & Flier, 2004). This endocrine tissue secretes different kinds of peptidic mediators known as adipokines. These have a secretion profile depending on the adipocyte hypertrophy, fulfilling numerous physiological functions, besides in the adipose tissue, in other target organs, including the brain, liver, muscle, vasculature, heart, pancreas and the immune system (Blüher & Mantzoros, 2015). There are adipokines with anti- and pro-inflammatory properties (Dahlman et al., 2012; Lehr et al., 2012a, 2012b; Pérez-Pérez et al., 2017). As anti-inflammatory adipokines are adiponectin or apelin, whereas in the pro-inflammatory ones can be found resistin, chemerin, visfatin, or the adipokine leptin, which is the objective of this review. Leptin, secreted in higher quantities in obese individuals, is the principal regulator of body weight and might be a factor in increasing MS risk in these subjects (Dahlman et al., 2012; Keyhanian et al., 2019) (Fig. 2).

Leptin, encoded by the *LEP* gene, can be sort out as a pro-inflammatory adipokine that has different immunological effects (Pérez-Pérez et al., 2017; Sánchez-Margalet et al., 2010). Leptin acts on specific hypothalamic nuclei inducing physiological net signal of satiety and generating energy expenditure, through inducing secretion of an anorexigenic signal (pro-opiomelanocortin) and suppressing an orexigenic one (neuropeptide Y) (Kwon et al., 2016). It can signal through six different splice variants of a receptor (LepRa, LepRb, LepRc, LepRd, LepRe, and LepRf), member of the Class I cytokine family (Tartaglia et al., 1995). Only the long form (LepRb) can signal intracellularly (Martín-Romero & Sánchez-Margalet, 2001; Sanchez-Margalet & Martin-Romero, 2001; Wada et al., 2014). The leptin receptor LepRb, when bound to leptin, is able to activate different signaling pathways through the autophosphorylation of “Janus Kinase 2” (JAK2). Once activated, it is capable, in turn, of phosphorylating different tyrosine residues of the leptin receptor (Tyr985, Tyr1077, and Tyr1138) that mediate intracellular signaling through different pathways. These intracellular pathways are JAK2/signal transducer activators of transcription 3 (STAT3), phosphatidylinositol 3-kinase (PI3K)/protein kinase B (Akt), extracellular signaling-regulated kinases (ERK), and signal transducer activators of transcription 5 (STAT5) (Martín-Romero & Sánchez-Margalet, 2001; Sanchez-Margalet & Martin-Romero, 2001; Wada et al., 2014). Furthermore, leptin influences both innate and adaptive immunities (Pérez-Pérez et al., 2017; Sánchez-Margalet et al., 2003). Regarding innate immunity, it activates the proliferation of monocytes/macrophages along with the production of pro-inflammatory cytokines, such as TNF-α, IL-6, and IL-12, among others (Sánchez-Margalet et al., 2010). It also affects neutrophils and NK cells (Ahmed et al., 2007; Tian et al., 2002) as well as dendritic cells (Mattioli et al., 2005). Concerning adaptive immunity, leptin stimulates the proliferation of naive T lymphocytes and promotes differentiation into Th1 lymphocytes that produce pro-inflammatory cytokines like INF-γ and IL-2. Additionally, it suppresses proliferation of regulatory T lymphocytes responsible for immunotolerance (Matarese et al., 2010; Versini et al., 2014), which has been suggested to be involved in the pathogenesis of MS (Matarese et al., 2008). In addition to all the effects mentioned above, leptin could play a significant role in the BBB since it alters permeability when the endothelium is damaged (de Candia & Matarese, 2018).

However, the majority of obesity cases result from the interaction of multiple genetic factors. Typically, obese individuals exhibit increased circulating leptin levels (Considine et al., 1996; Maffei et al., 1995a, 1995b), due to resistance to the effects of leptin (Schwartz et al., 1996). Consequently, treatment with leptin is often ineffective. Leptin, a 146-amino acid (16 kDa) polypeptide hormone (Zhang et al., 1994), shares similarities with members of the long-chain helical cytokine family (including IL-6, IL-11, IL-12, Leukemia inhibitory factor (LIF), granulocyte-colony stimulating factor (G-CSF), Ciliary neurotrophic factor (CNTF), and oncostatin M) and is primarily produced by adipose tissue (Maffei et al., 1995a, 1995b) It belongs to the class I long-chain helical cytokines and signals by interacting with a class I cytokine receptor (Ob-R), expressed in various tissues and cells (Tartaglia et al., 1995) (Pérez-Pérez et al., 2017; Sánchez-Margalet et al., 2003, 2010). Leptin levels correlate with adipose mass, resulting in higher leptin levels in obese individuals compared to leaner counterparts (Frederich et al., 1995; Maffei et al., 1995a, 1995b) Additionally, leptin production is regulated by different hormones, such as insulin (Pérez-Pérez et al., 2013; Wabitsch et al., 1996) or estrogen, potentially explaining the higher leptin levels found in women (Shimizu et al., 1997).

**Fig. 3:**
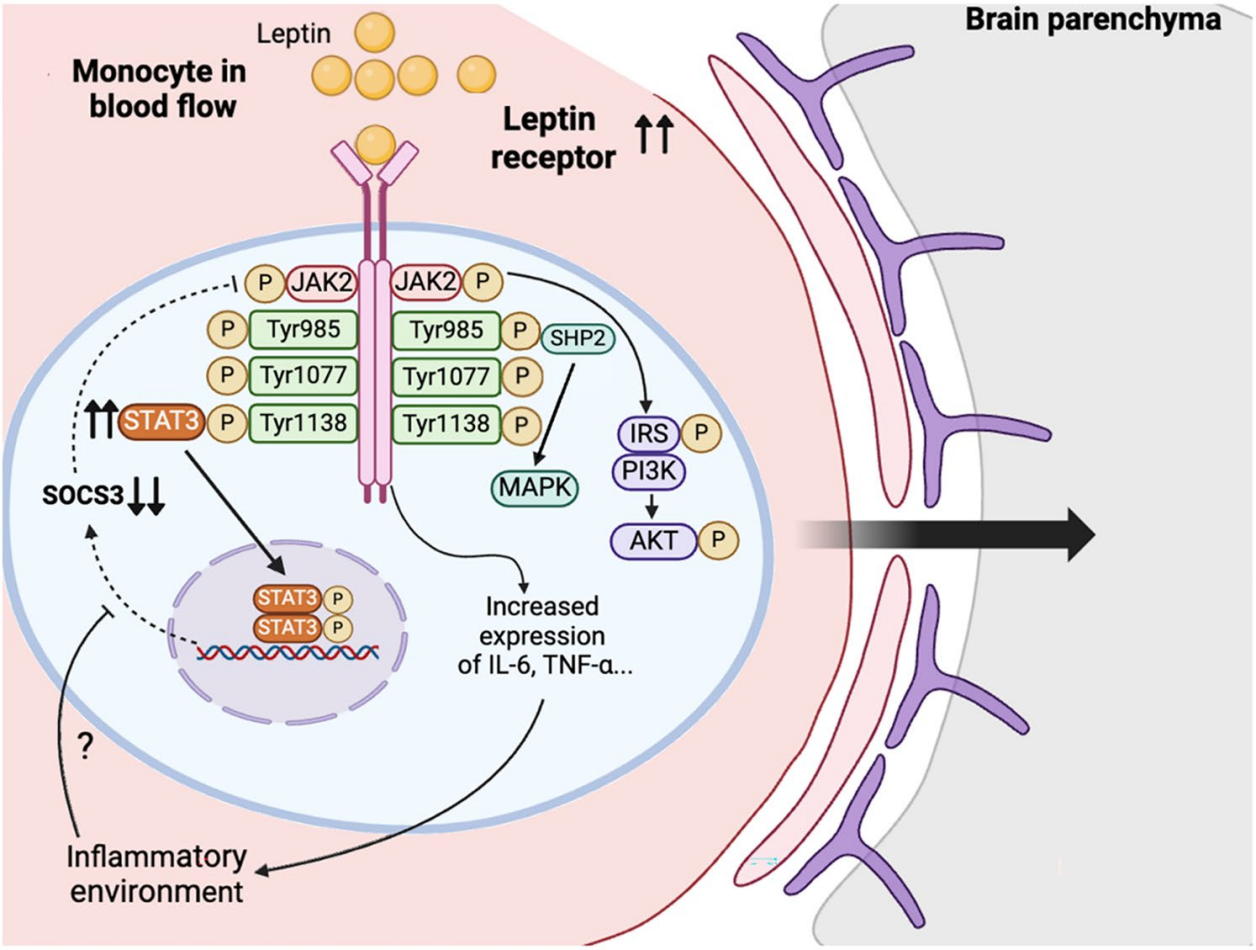
The leptin receptor on infiltrating monocytes is activated by leptin, which is present at elevated levels, particularly in obese individuals. Leptin receptor expression is increased in individuals with relapsing multiple sclerosis (MS), and this receptor is capable of initiating several intracellular signaling pathways, including JAK2/STAT3, PI3K/Akt, and ERK. Activation of the leptin receptor leads to an increased expression of pro-inflammatory cytokines, such as IL-6 and TNF-α, as well as enhanced phosphorylation of STAT3 in relapsing MS patients. However, SOCS3, a downstream regulator of the leptin receptor and JAK2/STAT3 pathway, is downregulated in MS. Under non-pathological conditions, SOCS3 is typically upregulated to negatively regulate leptin receptor signaling. The mechanisms underlying the reduced expression of SOCS3 in MS remain unclear. Finally, these infiltrating monocytes cross the blood–brain barrier to enter the central nervous system parenchyma, where they differentiate into macrophages

Leptin acts centrally as a satiety factor, inducing anorexigenic signals and inhibiting the production of orexigenic neuropeptides in the nucleus arcuate (Cowley et al., 2001). Nevertheless, leptin has various actions connecting metabolism with the immune system (Pérez-Pérez et al., 2017). It affects the innate immune system by activating the proliferation of monocytes/macrophages and inducing the production of pro-inflammatory cytokines, such as TNF-α, IL-6, and IL-12 (Loffreda et al., 1998) (Fig. 3). Leptin also acts on neutrophils and NK cells, where the leptin receptor is expressed—specifically, the short form in neutrophils (Ob-Ra) (Bruno et al., 2005; Zarkesh-Esfahani et al., 2004), and both the short and long forms in NK cells (Zhao et al., 2003). Furthermore, leptin influences the adaptive immune system by stimulating the proliferation of T lymphocytes and promoting differentiation toward the Th1 phenotype, resulting in the production of pro-inflammatory cytokines, such as INF-γ and IL-2 (Martín-Romero et al., 2000). Simultaneously, leptin suppresses the proliferation of Treg cells (Procaccini et al., 2010).

## Leptin and Multiple Sclerosis

Monocytes are one of the components of the innate immune system capable of crossing the blood–brain barrier (BBB) when compromised, as observed in autoimmune diseases like multiple sclerosis (MS). Notably, obesity has been shown to exacerbate the experimental autoimmune encephalomyelitis (EAE) mouse model of MS, leading to BBB disruption (Stampanoni Bassi et al., 2020), with leptin potentially contributing to this effect. The leptin receptor is expressed in human peripheral blood mononuclear cells (Martín-Romero & Sánchez-Margalet, 2001; Martín-Romero et al., 2000; Sanchez-Margalet & Martin-Romero, 2001) including monocytes (Santos-Alvarez et al., 1999) and elevated expression has been identified on monocytes of people with relapsing–remitting MS (RRMS) compared to those in remission or healthy controls (Frisullo et al., 2007). Furthermore, researchers observed increased p-STAT3 and decreased suppressor of cytokine signaling 3 (SOCS3) expressions in monocytes of relapsing people with MS with higher serum leptin levels than controls. These findings suggest a potential role for leptin signaling in the exacerbation of MS in relapsing patients, making leptin signaling a plausible target in the fight against MS (Fig. 3). Besides, leptin may contribute to the development of MS via oxidative stress as recently reviewed (Tanaka & Vécsei, 2020).

As Leptin can induce proliferation and phagocytic activity in human macrophages, as well as secretion of pro-inflammatory cytokines, such as TNF-α, IL-1β, and IL-6 (Dayakar et al., 2016), besides, in animal models, leptin can induce the expression of IL-6, IL1-β, and TNF-α in microglia (Lafrance et al., 2010; Pinteaux et al., 2007; Tang et al., 2007), a CNS-specific macrophage-like cell, responsible for survey the CNS parenchyma. Moreover, leptin, through its signaling pathways and in the context of MS, seems to have a pro-inflammatory role, which aggravate disease progression: in the context of EAE of the mouse model of MS, leptin administered exogenously may be able to aggravate such state, whereas starvation, which produces a significant decrease in leptin levels, may alleviate it (Lord et al., 1998). Similarly, administration of leptin to mice without leptin deficiency but susceptible to experimental autoimmune encephalomyelitis (EAE) worsens the disease course, while the administration of anti-leptin receptor antibodies ameliorates it (Matarese et al., 2001). Furthermore, the EAE mouse model has demonstrated in situ leptin production in inflammatory infiltrates and neurons, occurring exclusively during acute/active phases of EAE. Conversely, starvation delayed disease onset and attenuated symptomatology (Sanna et al., 2003). Corresponding results in humans have been observed. In people with relapsing–remitting multiple sclerosis (RRMS), increased levels of leptin in cerebrospinal fluid and blood serum have been demonstrated (Lock et al., 2002). Transcriptional analysis of leptin expression at sites of inflammation in MS brains showed elevated levels of leptin. More recently, an increased risk of MS was correlated with obesity and leptin in young people (Biström et al., 2021; Marrodan et al., 2021; Stampanoni Bassi et al., 2020). Similarly, serum leptin levels decreased in patients with secondary progressive MS (SPMS) who did not experience disease progression (Angelucci et al., 2005). Moreover, leptin levels may be a marker of activity in treated with interferon-beta (Batocchi et al., 2003).

Without considering obesity, no relationship with body mass index (BMI) was found. In a cross-sectional study comparing leptin serum levels between people with MS and a control group, a significant increase in leptin levels was described in individuals with MS, accompanied by a decrease in orexin-A and TGF-β (Moharami et al., 2022). However, a different study reported different results, with significantly lower blood leptin levels in the case group compared to the control group (Cinkir et al., 2021). No effect on leptin levels under dimethyl fumarate treatment was found, both in a longitudinal and a cross-sectional study, between MS-affected and control groups (Baharnoori et al., 2021) Similarly, no effect on leptin levels for MS risk was identified, although there was an effect on MS risk combined with low vitamin D levels in a Mendelian randomization study (Harroud et al., 2021).

Overall, this may suggest a regulatory role of leptin, where increased levels, as observed during obesity, tip the balance toward an exacerbation of the immune system and a loss of immune self-tolerance, potentially worsening MS progression. However, further clinical investigation in this field is necessary to clarify the role of obesity and leptin in MS, as the underlying mechanisms remain unclear.

## Dietary Interventions in Obese Patients with Multiple Sclerosis

It is well known that individuals with multiple sclerosis (MS) exhibit poorer dietary habits than their healthy counterparts, potentially contributing to observed cases of overweight and obesity among some patients. Additionally, studies have indicated that individuals with diets high in saturated fats are three times more likely to experience disease relapses, whereas diets rich in vegetables may reduce this risk. Hence, specific dietary habits appear to be associated with the progression of MS (Mische & Mowry, 2018).

The Mediterranean diet, renowned for its positive effects on cardiovascular health, has been examined in a large-scale study involving thousands of people with MS. Over 50% of these patients were found to have cardiovascular diseases, which correlated with a poorer prognosis and increased disability in walking, as indicated by higher scores on the Patient-Determined Disease Scale (PDDS). Elevated concentrations of triglycerides and low-density lipoprotein (LDL) in the blood were also linked to a greater number of lesions on T2-weighted magnetic resonance imaging, potentially resulting from micro-ischemic infarcts rather than typical MS-related lesions. However, it remains unproven that the Mediterranean diet, while beneficial for vascular pathologies, improves the Expanded Disability Status Scale (EDSS) or the Fatigue Severity Scale (FSS) (Mische & Mowry, 2018). Notably, many people with MS have experienced a clinical improvement with a low-fat diet (Yadav et al., 2016; Zhang et al., 2000). Although its impact on the prognosis or clinical-radiological activity of the disease has not been conclusively demonstrated, the low-fat diet proves useful for weight loss, reducing fatigue, and lowering LDL and blood cholesterol levels. It is plausible that the alleviation of fatigue in this context is more closely linked to a lower body mass index (BMI) than to the diet itself (Mische & Mowry, 2018). Increased sodium intake has been observed to worsen the severity of the disease in experimental autoimmune encephalomyelitis (EAE) models (Kleinewietfeld et al., 2013). In contrast, high-sodium diets do not seem to positively influence the course of MS in human studies (Cortese et al., 2017; Mische & Mowry, 2018).

Numerous experimental studies on EAE have consistently shown that calorie-restricted diets can mitigate the risk of disease onset, severity, neurodegeneration, inflammation, and increase survival rates in mice (Piccio et al., 2008). Various mechanisms have been proposed to explain how intermittent fasting, daily fasting, and calorie restriction can enhance the clinical course of the disease (Choi et al., 2016; Kafami et al., 2010). These dietary interventions regulate immune function by decreasing the secretion of interferon-gamma (IFN-γ), tumor necrosis factor-alpha (TNF-α), leptin, and interleukin-6 (IL-6), while elevating concentrations of IL-10, thereby reducing inflammation, demyelination, and MS-related axonal lesions.

Both daily and intermittent calorie restrictions throughout the week have been deemed safe for weight loss in people with MS. Additionally, these approaches help prevent cardiovascular events and enhance the emotional well-being of individuals with MS. Several studies have suggested that, particularly in the early stages of the disease before physical disability sets in, the emotional state of patients is one of the most crucial symptoms associated with later disability. Thus, even if improvements in the clinical and radiological condition are not achieved, weight loss through calorie restriction can alleviate symptoms, offering emotional relief to the patient (Fitzgerald et al., 2018).

The positive effects of these dietary interventions may be partly mediated by the improvement in leptin resistance and, consequently, the reduction in leptin levels, countering inflammation in MS, as previously suggested in diabetes and other complications associated with obesity (Montserrat-de la Paz et al., 2021; Pérez-Pérez et al., 2020a, 2020b).

## Final Remarks

Numerous factors contribute to chronic inflammation in the context of obesity, with adipokines, particularly leptin, emerging as the connection between obesity and immunity. Leptin, among its various functions, supports the in vitro survival of multiple immune cells implicated in the pathogenesis of MS. Additionally, leptin promotes inflammatory responses by increasing the proliferation of, among others, Th1 lymphocytes, while simultaneously reducing the number of Treg cells. Most studies have been conducted in animal models and studies in humans are scarce. Nevertheless, clinical studies seem to associate leptin levels with the disease (Table 1).

**Table 1:** Role of leptin in MS and clinical studies

Leptin levels	Effects on MS	MS phenotype	References
Increased blood leptin and/or leptin receptor levels	Disease activity	RRMS	(Cinkir et al., 2021; Frisullo et al., 2007; Marrodan et al., 2021; Moharami et al., 2022; Stampanoni Bassi et al., 2020)
Increased leptin levels in CSF	Disability	RRMS	(Stampanoni Bassi et al., 2020)
Low leptin levels	Lack of progression	SPMS	(Angelucci et al., 2005)
Nutritional intervention that may lower leptin levels	Possible less progression	PMS, RRMS	(Fitzgerald et al., 2018; Mische & Mowry, 2018; Yadav et al., 2016; Zhang et al., 2000)

As can be assessed in the Table, the low number of clinical studies is a clear limitation. Nevertheless, other clinical studies are in progress to investigate the role of leptin in MS and the response to treatment or nutritional intervention: EudraCT Number: 2013-004450-21; ClinicalTrials.gov: NCT02064816, NCT02411838, NCT03539094, NCT04593082, NCT02647502, NCT05327322, and NCT01067573.

Both the Mediterranean diet and caloric restriction represent safe methods for weight loss in obese individuals with MS, displaying promising outcomes, potentially by improving leptin resistance in the hypothalamus and reducing circulating levels. However, robust evidence supporting direct benefits is still lacking (Table 1).

In summary, scientific evidence suggests that obesity serves as a risk factor for MS, with this association becoming more pronounced when obesity occurs during critical ages between 12 and 18 years. Higher concentrations of pro-inflammatory adipokines, including leptin, have been observed in people with MS compared to healthy individuals. The relationship between clinical-radiological activity throughout the disease course and leptin concentrations remains unclear. Further studies are warranted to validate leptin concentration as a biomarker or potential therapeutic target.

## Data Availability

No datasets were generated or analyzed during the current study.
